# Food safety and handling knowledge and practices among university students of Bangladesh: A cross-sectional study

**DOI:** 10.1016/j.heliyon.2022.e11987

**Published:** 2022-11-30

**Authors:** Md. Nazrul Islam, Hussein F. Hassan, Md. Bony Amin, Felix Kwashie Madilo, Md. Ashiqur Rahman, Md. Raisul Haque, Md. Aktarujjaman, Nawshin Farjana, Nitai Roy

**Affiliations:** a Department of Post-Harvest Technology and Marketing, Faculty of Nutrition and Food Science, Patuakhali Science and Technology University, Patuakhali 8602, Bangladesh; b Department of Natural Sciences, Lebanese American University, Beirut, Lebanon; c Faculty of Nutrition and Food Science, Patuakhali Science and Technology University, Patuakhali 8602, Bangladesh; d Department of Food Science and Technology, Faculty of Applied Science and Technology, Ho Technical University, Volta Region, Ho, Ghana; e Department of Fisheries and Marine Bioscience, Jashore University of Science and Technology, Jashore, 7408, Bangladesh; f Department of Biochemistry and Food Analysis, Faculty of Nutrition and Food Science, Patuakhali Science and Technology University, Patuakhali 8602, Bangladesh

**Keywords:** Knowledge, Practices, Food safety, Bangladesh, University students, Questionnaire

## Abstract

Our study aimed to examine the practices and knowledge of food handling and safety among 1534 university students in Bangladesh (mean age 22.09 ± 1.78), as well as the relationship between these factors and their academic and demographic backgrounds. Participants in this study were undergraduate and graduate students from four public universities in Bangladesh from different religions, income levels, years and majors of study, residential areas, living alone or not, and whose mothers are working or non-working. The questionnaire included 14 questions on food handling practices and 16 on knowledge. Questions were related to food preparation, hygiene, cross-contamination, and storage. The overall mean score for food handling practices was 34.9% (SD = 13.7), while that of knowledge was 41.8% (SD = 16.5). Female students, those from food-related majors, and those engaged in cooking activities scored significantly higher in the knowledge and practice sections (*p < 0.05)*. Students who lived with their families performed significantly better on the knowledge parts, while those who shared a home with roommates in mess performed significantly better on the practice part (*p < 0.05)*. Students having housewife mothers, personal poisoning experience, and continuous involvement in food purchasing scored significantly higher (*p < 0.05)* in the practices section but not in the knowledge one. On the other hand, students living in urban areas scored significantly higher (*p < 0.05)* in the knowledge section but not in the practices one. Our results highlight the importance of educational interventions and initiatives to enhance food safety awareness among Bangladeshi university students.

## 1 Introduction

The global public health concern is foodborne infections, even in nations with sophisticated food safety systems, such as ^“^farm-to-fork^”^ in Europe and ^“^farm-to-table^”^ in the USA (Morris, 2011; Lazou et al., 2012). Though it is difficult to measure the prevalence of foodborne diseases worldwide, it is thought that 1.6 million people each year pass away from acute gastroenteritis, primarily brought on by infected water and food. In the USA, each year, 1/3^rd^ of people may get a foodborne illness. This percentage is increasing due to the globalized food trade, increasing tourism, mass production, and industrialized animal production, among others (Hassan and Dimassi, 2014). Therefore, safe food purchase, cooking, preparation, and handling in households are key to reducing the prevalence of foodborne illnesses (Redmond and Griffith, 2003; Kennedy et al., 2005).

Mishandling of foods among young male adults (less than 30 years old) has been commonly reported (Li-Cohen and Bruhn, 2002; Medeiros et al., 2004; McArthur et al., 2007). Despite the fact that this age group is not considered ^“^at-risk^”^ for foodborne illnesses, their poor hygienic practices have serious ramifications when they begin to provide care for other at-risk family members (children, the elderly, and pregnant women) (Abbot et al., 2009; Byrd-Bredbenner et al., 2008). A plethora of studies on food safety knowledge and practices were conducted on youth, such as in Saudi Arabia (Sharif and Al-Malki, 2010), USA (Byrd-Bredbenner et al., 2007; Morrone and Rathburn, 2003), Turkey (Sanlier, 2009), Spain (Garayoa et al., 2005), Greece (Lazou et al., 2012), Lebanon (Hassan and Dimassi, 2014), Iran (Eslami et al., 2015), Canada (Majowicz et al., 2015), Bulgaria (Stratev et al., 2017), Pakistan (Zeeshan et al., 2017), Ethiopia (Azanaw et al., 2019), Jordan (Osaili et al., 2011), Kuwait (Ashkanani et al., 2021), Oman (Al Makhroumi et al., 2022), Serbia (Vuksanović et al., 2022), and Iraq (Muhyaddin et al., 2022).

Determining the actual level of food safety knowledge and practices is the starting point to improve this level among any group via educational programs. In other words, identifying the demographic factors of the handlers having the poorest knowledge of food safety is the foundation of any effective educational program. Numerous studies have documented the effect of different demographic characteristics, such as gender, income, education level, age, major of study, and residential location, on food handling knowledge and practices (Sanlier, 2009; Hassan and Dimassi, 2014; Ovca et al., 2014). However, these studies showed mixed results because of variations in population characteristics, design of studies, and questions of the survey.

Foodborne diseases or illnesses and other food safety hazards are prevalent in Bangladesh due to the high population density, poor water, sanitation, and hygiene (WASH) facilities, and unfledged infrastructure (Noor and Feroz, 2016). As a result, each year nearly 30 million individuals suffer from foodborne infections in Bangladesh (Khairuzzaman et al., 2014). In particular, diarrheal illnesses are the most prevalent foodborne illnesses, in addition to hepatitis and enteric fever as common cases (Food and Agricultural Organization of the United Nations, 2016; Suman et al., 2021).

Al Mamun et al. (2013) investigated food safety knowledge and awareness among school-based street food vendors in Bangladesh, whereas Al Banna et al. (2021) evaluated factors influencing food safety knowledge and behaviors among meat processors. Additionally, knowledge and practices were assessed among fish farmers/restaurants, food handlers, chotpoti vendors, street food vendors, and chicken vendors in Bangladesh by Hashanuzzaman et al. (2020), Siddiky et al. (2022), and Hossen et al. (2020), respectively. Additionally, Tarannum (2021) investigated the practices, attitudes, and knowledge of food handlers in Bangladeshi households about food sanitation. As far as our search is concerned, there has not been any previous data on food safety knowledge and practices among students in Bangladeshi universities; therefore, our study assessed self-reported practices and knowledge related to food safety among four public universities in Bangladesh. Another important goal was to evaluate the impact of various demographic traits on handling behaviors and knowledge of food safety. The findings from our study can be used to set legislation, promote correct practices, design effective interventions to improve knowledge, and change false beliefs and practices related to food handling.

## 2 Materials and methods

### 2.1 Study subjects

Between January and March 2022, a cross-sectional study at four public universities in Bangladesh was carried out. These universities were Patuakhali Science and Technology University (PSTU), Jashore University of Science and Technology (JUST), Hajee Mohammad Danesh Science and Technology University (HSTU), and Islamic University (IU). The selection of universities considered those offering bachelor's degrees in both food-related (nutrition and food science, applied food technology and nutritional science, food processing and preservation technology) and non-food-related majors of study (arts, basic sciences, business studies, technology, and engineering). The inclusion criteria for the study include the following: Bangladeshi university students (both genders) registered at one of the selected universities from different majors of the study (food-related or non-food-related) and levels of the study (bachelor or master).

A total of 1650 participants were randomly selected using random number table from each major of the study, where 436 participants were from food-related majors of study, and the rest (1214 participants) were from non-food-related majors. The questionnaire was distributed to ensure diversity in terms of gender and major of study. Before filling out the questionnaire, an oral explanation of the study content, objectives, and the study protocol was given to the class teacher/instructor for initial approval. After getting the approval, the researchers met the students. They informed them about the importance, objectives and protocol of the study, and those who volunteered to take part in the study gave their written approval. Then, questionnaires were supplied to all the students in the selected classrooms so that they could be filled at the end of the sessions.

No other rewards were provided since the participation was completely voluntary and anonymous. Inappropriately filled or incomplete questionnaires were excluded from the study. Hence, out of 1650 filled questionnaires, only 1534 (92.96% response rate) were valid (396 from food-related majors and 1138 from non-food-related majors). Ethical approval was granted by the ethical committee of the department of Biochemistry and Food Analysis at Patuakhali Science and Technology University (PSTU) (approval number: BFA: 10/12/2021:04).

### 2.2 Questionnaire

To evaluate food handling and safety knowledge and practices among Bangladeshi university students, a questionnaire was developed by including questions collected from updated, valid, and reliable instruments produced by previous studies (Hassan et al., 2014; Hassan et al., 2018; Chuang et al., 2021; Lazou et al., 2012; Byrd-Bredbenner et al., 2007; Haapala and Probart, 2004; Ovca et al., 2014; Osaili et al., 2011). In addition, some questions related to geographical location, culture and eating habits in Bangladesh were modified. The questionnaire was piloted among 45 students to determine whether the wording was precise and appropriate and determine how long it would take to complete. Modifications were made based on the results of the pilot research.

The final questionnaire is divided into four sections: an introduction, demographic data, handling procedures for food safety, and food safety knowledge. The first part included a short introduction to the objectives of the study. The second part focused on demographic characteristics, including age, gender, religion, current educational status, residential status, monthly income (BDT), mother employment status, cooking habits, previous personal food poisoning experience, involvement in food purchasing, etc. The third part comprises the food safety practices section containing 14 questions that were divided into 4 subsections: food microbiology and cross-contamination practices (4 items), food preparation and cooking practices (4 items), food storage and chilling practices (3 items), and cleaning and hygiene practices (3 items). Most of the questions were multiple-choice based. Part 4 was designed to assess the knowledge on food safety, which included 16 questions divided into four subsections: food microbiology and cross-contamination (4 items), food preparation and cooking knowledge (5 items), food storage and chilling (4 items), and cleaning and hygiene (3 items). Most of the questions in this section were multiple-choice based. The questionnaire took approximately 20 min to be filled.

### 2.3 Statistical analysis

Statistical software, SPSS version 28.0, was used to analyse the data acquired. Simple descriptive tests were used to observe the frequency, percentages, mean, standard deviation, and standard error. Each correct answer to each multiple-choice question was given a score of 1, while 0 for all wrong answers. Thus, food safety practices section score varies from 0 to 14 and the knowledge section from 0 to 16. Then, normality was checked for each variable with the dependent variable to observe the distribution of scores for each category. Due to skewed distribution, non-parametric tests (Wallis H test and Mann-Whitney U test) were performed to observe the differences of the mean sum of the correct responses of knowledge (16 questions) and practice (14 questions) sections within demographics. All tests were two-sided and done with 95% confidence intervals. Tests were considered significant when the *p*-value was found to be less than 0.05.

## 3 Results and discussion

A total of 1534 valid questionnaires filled by undergraduate and graduate students were analyzed, among which 49% were females, and 51% were males. In addition, 25.8% of students were from food-related majors and 74.2% from non-food-related majors (basic sciences, arts, business, technology, and engineering). The mean student age was 22.09 (SD = 1.78). The majority of the undergraduate students were in 1^st^ year (36%) while the postgraduate students formed 8% of the total population. About 9% of the participants lived with their parents. However, only 8% of the subjects answered that they cook all the time, and only 17% of the participants had a working mother (Table 1).

**Table 1:** Socio-demographic characteristics of the study population (N = 1534).

Demographic variables	Category	Count	%
Major of study	Food-related	396	25.8
	Non-food-related	1138	74.2
Age (Years)	18 to 20	340	22.2
	21 to 23	831	54.1
	Above 23	363	23.7
Gender	Male	776	50.6
	Female	758	49.4
Religion	Muslim	1298	84.7
	Hindu	223	14.5
	Other^a^	13	0.8
Current educational status	B.Sc. 1st year	547	35.7
	B.Sc. 2 nd year	373	24.3
	B.Sc. 3rd year	268	17.5
	B.Sc. 4th year	221	14.4
	Masters'	125	8.1
Residential status	With Family	135	8.8
	With Friends/Roommates in Hall	1186	77.3
	In mess with roommates	213	13.9
Place of residence	Rural	814	53.1
	Urban	720	46.9
Father's education	No formal education	111	7.2
	Primary	126	8.2
	Secondary	312	20.3
	Higher secondary	359	23.5
	Bachelor and/or above	626	40.8
Mother's education	No formal education	131	8.5
	Primary	206	13.4
	Secondary	495	32.3
	Higher secondary	409	26.7
	Bachelor and/or above	293	19.1
Monthly income (BDT)	Up to 15000	419	27.3
	16000 to 30000	696	45.4
	Above 30000	419	27.3
Mother employment status	Employment/works	258	16.8
	Housewife	1276	83.2
Cooking habit	Yes, all time	126	8.2
	Yes, sometimes	655	42.7
	Yes, rarely	486	31.7
	Never	267	17.4
Personal food poisoning experience	Yes	986	64.3
	No	548	35.7
Involvement in food purchasing for personal or family	Yes, all times	139	9.1
	Yes, sometimes	621	40.5
	Yes, rarely	417	27.2
	Never	357	23.2

Note: a = Buddhist and Christians, Exchange rate was 15000 BDT = 174 USD.

Table 2 displays the mean score for the practices and knowledge and the significant levels for each variable. The overall mean score for best practices in food handling was 34.9% (SD = 13.7). For the food handling part, female students and students living with their families scored significantly better than their male counterparts living with friends or roommates. Older students (above 23 years old) with food-related majors performed significantly better than younger students from non-food majors (*p* < 0.001 and p = 0.001, respectively). The participants with working mothers scored higher, with the significant different at (*p* = 0.048). Students with personal food poisoning experience and full involvement in food purchasing scored significantly higher (*p* = 0.002). On the other hand, the effects of religion, maternal education, and monthly income were not significant (*p* > 0.05), meaning that they have no influence on food safety knowledge or handling practices.

**Table 2:** Mean scores of food handling practice and food safety knowledge sections per demographic characteristics.

Demographic variable		Food handling practices			Food safety knowledge		
		Mean	SD	*p*-value	Mean	SD	*p*-value
**Major of study**							
	Food related	40.17	13.31	<0.001	47.32	14.93	<0.001
	Nonfood related	33.10	13.38		39.85	16.52	
**Age (Years)**							
	18 to 20	35.46	13.26	0.001	41.36	15.81	0.919
	21 to 23	35.77	13.72		41.87	16.27	
	Above 23	32.49	13.85		41.96	17.44	
**Gender**							
	Male	33.94	13.71	0.007	40.70	17.47	0.005
	Female	35.93	13.64		42.88	15.25	
**Religion**							
	Muslim	34.89	13.62	0.983	41.87	16.58	0.741
	Hindu	35.14	14.37		41.45	15.71	
	Other^a^	34.07	12.08		38.46	16.11	
**Year of study**							
	B.Sc. 1st year	34.85	13.23	0.003	40.39	16.11	0.062
	B.Sc. 2 nd year	37.17	14.46		41.92	16.00	
	B.Sc. 3rd year	33.64	13.79		41.81	16.40	
	B.Sc. 4th year	34.49	14.07		42.08	16.30	
	Masters'	32.06	11.74		46.80	18.63	
**Residential status**							
	With Family	37.99	14.33	0.003	42.87	16.29	<0.001
	With Friends/Roommates in Hall	34.41	13.65		40.77	15.98	
	In mess with roommates	35.85	13.38		46.71	18.15	
**Residential area**							
	Rural	35.21	13.49	0.434	40.69	15.73	0.007
	Urban	34.60	13.96		43.00	17.15	
**Mother's education**							
	No formal education	34.19	13.29	0.747	39.55	16.99	0.113
	Primary	34.60	14.59		39.71	15.58	
	Secondary	34.50	13.66		42.94	16.72	
	Higher Secondary	35.50	13.60		42.02	16.62	
	Bachelor and/or above	35.37	13.54		41.92	15.95	
**Monthly income (BDT)**							
	Up to 15000	34.78	13.56	0.509	40.32	15.13	0.062
	16000 to 30000	34.55	13.57		41.65	16.49	
	Above 30000	35.68	14.10		43.45	17.49	
**Mother employment status**							
	Employment/works	33.25	13.81	0.048	40.21	16.83	0.079
	Housewife	35.26	13.67		42.09	16.35	
**Cooking habit**							
	Yes, all time	32.82	14.49	0.007	40.03	15.64	<0.001
	Yes, sometimes	35.80	13.63		41.86	15.92	
	Yes, rarely	35.36	13.56		43.80	16.63	
	Never	32.96	13.58		38.72	17.27	
**Personal food poisoning experience**							
	Yes	35.76	13.85	0.002	42.02	15.92	0.189
	No	33.42	13.34		41.34	17.36	
**Involvement in food purchasing for personal or family use**							
	Yes, all times	34.99	14.03	0.020	41.01	16.10	0.874
	Yes, sometimes	35.15	14.06		41.82	16.88	
	Yes, rarely	33.38	13.40		41.74	16.29	
	Never	36.29	13.20		42.05	16.04	
Total		34.92	13.71		41.78	16.45	

Note: a = Buddhist and Christians.

The overall mean score of food safety knowledge was 41.8% (SD = 16.5). Female students and those majoring in food-related fields of study scored significantly better than males and students from non-food majors (*p* = 0.005 and *p* < 0.001, respectively). In addition, subjects from urban areas and those who cook all the time had significantly higher score (*p* = 0.007 and *p* < 0.001, respectively). On the other hand, age, religion, year of study, maternal education and employment status, monthly income, personal food poisoning experience, and involvement in food purchasing were not significant (*p* > 0.05).

The responses reported in our study identified poor levels of food safety knowledge (41.8%) and implementation of food handling practices (34.9%). This poor food safety awareness was as well reported in the literature (Byrd-Bredbenner et al., 2007; Abbot et al., 2009; Garayoa et al., 2005; Osaili et al., 2011; Lazou et al., 2012; Unklesbay et al., 1998; Sharif and Al-Malki, 2010; Hassan and Dimassi, 2014). For instance, among university students in Greece, the United States, and Lebanon, the mean scores for food safety knowledge were 60, 60, and 54%, respectively, whereas the mean scores for food handling procedures were 44, 50, and 49%, respectively (Lazou et al., 2012; Hassan and Dimassi, 2014; Byrd-Bredbenner et al., 2007).

Students from food-related majors reported significantly (*p* < 0.001) higher scores on practices (40.2%) and knowledge (47.3%) scores (Table 2). This can be attributed to food safety, hygiene, and microbiology modules in the food-related major curricula. A similar conclusion was reported by Hassan and Dimassi (2014); Byrd-Bredbenner et al. (2007); Osaili et al. (2011); Garayoa et al. (2005); Unklesbay et al. (1998); Sharif and Al-Malki (2010).

Female students showed significantly higher scores than their male counterparts with regard to food handling practices (35.9%; *p* = 0.007) and food safety knowledge (42.9%; *p* = 0.005) (Table 2). This may be explained by the fact that women are typically in charge of maintaining the cleanliness and hygiene of the kitchen throughout East Asia, especially in Bangladesh. This goes in line with previous studies (Unklesbay et al., 1998; Lazou et al., 2012; Byrd-Bredbenner et al., 2007; Hassan and Dimassi, 2014).

In terms of practices and knowledge, students who lived with their families performed better than those who lived with friends or roommates (Table 2), and the difference was statistically significant (p = 0.003 and p 0.001, respectively). Hassan and Dimassi reported the same observation (2014). This could be because when students live with their family, a more seasoned individual (the mother in the case of Bangladesh) will prepare the meals, leading to more standardized food handling and an opportunity for the student to learn more about food safety. Participants with working mothers scored less in both practice (33.3%) and knowledge (40.2%) questions, yet the difference was borderline significant (*p* = 0.048) for the practices only. The reason could be the fact that, in general, working mothers are usually educated, and therefore, they spend less time on food preparation compared to housewives, resulting in poorer food safety knowledge and food handling practices.

Concerning food handling practices, scores for each question are presented in Table 3. For instance, among the correct practices, only 26.7% of participants reported using another chopping board when switching from cutting meat to cutting vegetables, while 45.8% reported washing the knife with soap and hot water when switching from raw meat to another food. Additionally, while only 26.4% of subjects with a wound on their hand reported handling food after wearing gloves, almost half of the respondents (49.7%) said washing their hands after touching their faces. On the other hand, only 50.8% rub their hands with soap for about 20 s when they want to wash their hands, and only 18.2% take off jewellery when preparing food. Surprisingly enough, while as low as 2.5% of participants reported checking the central temperature of the cooking pot to verify that food is sufficiently cooked, 6.8% of them thawed raw meat in the refrigerator, and 10% reported throwing meat away when it thaws and feels warm when the electricity goes off. This poor knowledge in food handling might be due to insufficient food safety and hygiene education on our tertiary education campuses.

**Table 3:** Score distribution to food handling practices questions.

Questions	Multiple-choice responses	Correct responses (%)
(1) You cut meat on a chopping board and now you wants to cut vegetables. Of the following, which one do you practice?	Use the board as it is.	4.0
	You wipe the board off with a paper towel/cloths	35.1
	Use the other side of the chopping board to cut vegetables	21.8
	**Use another chopping board to cut vegetables**^d^	**26.7**
	Don't know	12.4
(2) When you cut raw meat and need to use the knife again, what do you do?	You reuse the knife as it is	4.2
	You rinse the knife with cold water	38.8
	You wipe the knife with a cloth/paper towel	11.2
	**You wash the knife with soap and hot water**^d^	**45.8**
(3) A refrigerator has three shelves, on which shelf do you place raw meat?	Top shelf	20.3
	Middle shelf	6.6
	**Bottom shelf**^d^	**61.7**
	Does not matter	11.4
(4) Do you handle food if you have a wound on the back of your hand?	Yes, as long as the wound has a bandage on it	25.6
	Yes, as long as the wound is not infected	17.7
	**Yes, as long as gloves are worn**^d^	**26.4** 30.3
	Not at all	
(5) How do you check that food is sufficiently cooked?	By seeing the food color/By taking taste	72.4
	Density of Juice content/concentration of food	18.5
	**By checking the central temp. of cooking pot**^d^	**2.5**
	Measuring the cooking time	6.6
(6) How long do you heat Leftover foods?	**Until they are boiling hot**^d^	**39.9**
	Heat it to the temperature you prefer	32.4
	Just until they are at least at room temperature or 250C	11.9
	Reheating is not necessary	3.3
	Don't know	12.5
(7) While washing your hands, how long do you rub them with soap?	10 s	25.0
	**20 s**^d^	**50.8**
	30 s	11.4
	40 s	4.0
	Don't know	8.8
(8) Do you take off the jewelry when preparing food?	**Yes**^d^	**18.2**
	No	27.6
	Yes, sometimes	13.7
	Not applicable	40.5
(9) Of the following, how do you thaw raw meat?	**Thaw In refrigerator**	**6.8**
	Thaw on chopping/cutting board (25 0 C/room temperature)^d^	13.6
	Thaw in cold water in sealed package/pot	41.2
	Thaw In running water	31.7
	Don't know	6.7
(10) In case your electricity went off and the meat, chicken, and/or seafood in your freezer thawed and felt warm, what do you do?	**Throw them away**^d^	**10.0**
	Cook them right away	28.7
	See how they smell or look before deciding what to do	44.7
	Immediately re-freeze until future consumption	16.6
(11) If your roommate or you are going to be several hours late for a hot meal, where do you leave the meal?	**Store it in the refrigerator and reheat it when the person is ready to eat it**^d^	**31.7**
	Store it in on the kitchen counter until the person is ready to eat it	36.2
	Store it in a warm oven until the person is ready to eat it Not reheat again	11.1
	Store it in a cool oven until the person is ready to eat it	18.2
	Don't know	2.8
(12) How do you wash your hands before starting preparing food or eating?	Cold Water only	6.8
	**Wash hand With soap/hand wash and cold water**^d^	**83.7**
	Wipe with a towel or dish cloth	6.1
	I don't clean them at all	3.4
(13) You wash fruits and vegetables by using:	Water and soap	6.8
	Hot water	11.0
	**We wash them under cold running water**^d^	**35.9**
	Using Normal water	46.3
(14) After touching which of the following do you wash your hands during the course of preparing food?	**Face**^d^	**49.7**
	Clean cooking utensils/cooking pot	33.6
	Clean utensils	5.3
	None of the above	11.4

d correct answer.

In addition, Table 4 presents the results of food safety knowledge of the participants. The table reveals that about 44% and 41.3% of participants knew that *Campylobacter* is most likely associated with raw meat/fish and that raw meat/fish is most likely to become contaminated with *Listeria*. Only 11.7% of students knew that teenagers are the least prone to get food poisoned, and 11.1% knew that foods are safe if cooked to an internal temperature of 74 °C. On the other hand, 31.9% of the respondents knew that the maximum fridge temperature is 4 °C, and 41.7% knew that freezing does not kill harmful germs in food.

**Table 4:** Score distribution to food safety knowledge questions.

Questions	Multiple-choice responses	Correct responses (%)
(15) Campylobacter bacteria are most likely associated with which food?	Canned food	14.5
	**Raw or undercooked meat/fish**^d^	**44.0**
	Fresh vegetables	4.5
	Don't know	37.0
(16) Which of the following is most likely to become contaminated with Listeria?	Canned food	13.1
	**Raw or undercooked meat/fish**^d^	**41.3**
	Fresh vegetables	5.9
	Don't know	39.7
(17) The microorganisms that cause most of food-borne illnesses are:	**Bacteria**^d^	**62.4**
	Fungi	14.0
	Viruses	5.5
	Parasites	5.5
	Don't know	12.6
(18) Which of these individuals are LEAST likely to get food poisoning?	Old people	38.3
	Pregnant women	25.6
	**Teenagers**^d^	**11.7**
	Don't know	24.4
(19) When is the best time to purchase frozen food when shopping?	At the beginning of the shopping time	9.4
	**At the end of the shopping time**^d^	**63.0**
	Whenever, does not matter	7.7
	Don't know	19.9
(20) All foods are considered safe when cooked to an internal temperature	54 °C	7.6
	60 °C	11.2
	66 °C	6.0
	**74 ° C**^d^	**11.1**
	Don't know	64.1
(21) Which is the safest way to get fried egg?	**Solid albumen and yolk**^d^	**43.2**
	Semi-solid albumen and yolk	18.6
	Solid albumen and semi-solid yolk	19.2
	Solid albumen and liquid yolk	7.8
	Don't know	11.2
(22) How to prevent salmonella poisoning?	**Fully heat food**^d^	**51.7**
	Freeze food for more than 3 days	10.3
	Those food will not safe for cooking	7.4
	Don't know	30.6
(23) People with which of the following symptoms should not cook for others?	**Diarrhea, Fever, Sore throat or Flu**^d^	**58.3**
	Skin allergies	16.0
	Headache	2.4
	All the Above	23.3
(24) What is the maximum refrigerators temperature should be to preserve the safety of foods?	-4 °C	35.7
	12 °C	4.6
	**4 °C**^d^	**31.9**
	Don't know	27.8
(25) What is the recommended temperature for freezers?	**-18°C**^d^	**45.7**
	0 °C	13.3
	18 °C	4.6
	Don't know	36.4
(26) You can get food poisoning from eating which of the following?	Fruits taken out of the refrigerator immediately	3.3
	Raw or undercooked eggs	12.5
	Raw or undercooked meat	17.9
	**Both B & C**^d^	**59.5**
	Others	6.8
(27) Freezing Kills harmful germs in food	Right	24.8
	**Wrong**^d^	**41.7**
	Don't know	33.5
(28) Which is the most important for preventing food poisoning?	Use detergent to disinfect kitchen countertop and stove weekly	17.5
	Avoid eating leftovers	19.2
	**Keep food refrigerated until it is time to serve them**^d^	**6.1**
	Washing hands properly before eating	8.2
	Don't know	49.0
(29) Of the following, which do you think is the correct way to wash dishes?	Soak in water, after several hours, wash with the same water using detergent/Ash/Soap	17.9
	Wash immediately after meal using detergent/Ash/Soap and wipe off	42.8
	**Wash immediately after meal using detergent/Ash/Soap and wipe off by towel**^d^	**27.1**
	Wash with automatic dish washer	12.2
(30) Which of the following scenario for cleaning kitchen counters and stoves are the best?	**Brush with Soap/detergent and water, then use sanitizer**^d^	**61.1**
	Using Sanitizer, then water	17.0
	Brush with water, then sanitizer	12.4
	Water, then drying	9.5

d correct answer.

A comparison between our study with similar questions from a developed country, Greece (Lazou et al., 2012), and a developing country, Lebanon (Hassan and Dimassi, 2014), presented in Table 5, reveals that Bangladeshi university students scored the least (20%) compared to the Greek (27%) and Lebanese (29%) students in the food storage practice, while they scored better (45%) than the Greek (37%) and Lebanese (39%) students in cross-contamination practice questions. For the cleaning and hygiene practice, Bangladeshi students scored (67%) compared to 68% in Greece and 61% in Lebanon. As for the overall mean for the food handling common practice questions (7 questions), our score was 44%, which is similar to that of Lebanon (43%) and Greece (44%).

**Table 5:** Comparison of food handling and safety practices and knowledge among Bangladesh, Lebanese and Greek university students.

		Best answer	Scores (%)		
			Bangladesh (our study)	Lebanon	Greece
**Food handling practice question**					
Cross-contamination	When you cut raw meat and need to use the knife again, what do you do?	You wash the knife with soap and water	46	74	67
	In the fridge (not freezer) of your house, where is the raw meat stored?	Lowest shelf	62	16	23
	If you have a sore on the back of your hand, do you prepare food?	Yes, but after you bandage the sore and wear a glove	26	27	20
	**AVERAGE**		**45**	**39**	**37**
Food storage					
	At home, how do you defrost frozen meat/chicken?	You leave it in the fridge for few hours	7	28	25
	If your roommate or family member is going to be several hours late for a hot meal, where do you leave the meal?	In the fridge	32	29	28
	**AVERAGE**		**20**	**29**	**27**
Cleaning and hygiene	How do you wash your hands before cooking or eating?	Soap and water	84	87	97
	When preparing food, you wash your hands after touching which of these?	Your face	50	34	39
	**AVERAGE**		**67**	**61**	**68**
**Food handling practice overall average**			**44**	**43**	**44**
**Food safety knowledge question**					
Food cooking	For a burger to be safe to eat, it needs to be cooked until its internal temperature reaches:	74**°**C	11	38	21
	How can a food be made safe if it has salmonella bacteria in it?	Cook it well	52	68	53
	**AVERAGE**		**32**	**53**	**37**
Food storage	During your supermarket shopping, when do you place refrigerated meat in your cart?	At the end of the shopping trip	63	60	55
	What is the recommended temperature for fridges?	4**°**C	32	53	44
	Freezing kills harmful germs in food	FALSE	42	64	78
	**AVERAGE**		**46**	**59**	**59**
**Food safety knowledge overall average**			**39**	**56**	**48**

With regards to food safety knowledge, Bangladeshi university students scored the least (32%), when compared to the Greek (37%) and Lebanese (53%) students. For cooking of food, Bangladeshi students again scored the least (46%) compared to the Greek (59%) and Lebanese (59%) students. The overall mean scores also indicated that Bangladeshi students’ score was 39%, which was the lowest when compared to Lebanon (56%) and Greece (48%) with respect to food safety knowledge (Table 5).

## 4 Concluding remarks

The poor food safety awareness reported by Bangladeshi university students results in an increasing intake of risky foods and, therefore, a higher likelihood of foodborne diseases. This poor awareness of food safety will contribute, in the long run, to a higher likelihood of foodborne illnesses in household settings, as university students will be at some point, food handlers and caregivers for their families. Information collected from this study has identified the urgent need for food safety education among youth, in high schools and universities, on proper temperature control, prevention of cross-contamination, proper food preparation practices, cleaning, sanitation, and hygiene. Higher academic institutions can be the correct place to intervene and reach out to the uneducated and the younger generation. Although its limitations related to the design as sampling was based on four universities only in Bangladesh, in addition to the fact that we used the questionnaire of other similar studies without validating it, our study gave considerable insights to the status of food safety knowledge and practices in Bangladesh.

## Declarations

### Author contribution statement

Md. Nazrul Islam: Conceived and designed the experiments; Performed the experiments; Contributed reagents, materials, analysis tools or data; Wrote the paper.

Hussein F. Hassan: Analyzed and interpreted the data; Contributed reagents, materials, analysis tools or data analysis tools or data; Wrote the paper.

Md. Bony Amin, Felix Kwashie Madilo: Analyzed and interpreted the data; analysis tools or data; Wrote the paper.

Md. Ashiqur Rahman, Md. Raisul Haque, Md. Aktarujjaman, Nawshin Farjana: Performed the experiments; Contributed reagents, materials, analysis tools or data.

Nitai Roy: Conceived and designed the experiments; Performed the experiments; Analyzed and interpreted the data; Contributed reagents, materials, analysis tools or data; Wrote the paper.

### Funding statement

This research did not receive any specific grant from funding agencies in the public, commercial, or not-for-profit sectors.

### Data availability statement

Data will be made available on request.

### Declaration of interest's statement

The authors declare no conflict of interest.

### Additional information

No additional information is available for this paper.

## Appendix A Supplementary data

Questionnaire (Bangla Version) .docx Questionnaire (Bangla Version) .docx

Questionnaire (English Version).docx Questionnaire (English Version).docx
