# Supplementary material for: Food safety and handling knowledge and practices among university students of Bangladesh: A cross-sectional study
Source: Heliyon. 2022 Nov 30;8(12):e11987. doi: 10.1016/j.heliyon.2022.e11987 (PMC9720042; doi:10.1016/j.heliyon.2022.e11987)
Supplement: Questionnaire (Bangla Version) .docx [file mmc1.docx]

| গবেষণা শিরোনামঃ  বাংলাদেশের বিশ্ববিদ্যালয়ের শিক্ষার্থীদের মধ্যে খাদ্য নিরাপত্তা **বিষয়ক** জ্ঞান এবং খাদ্য **ব্যবস্থাপণার** অনুশীলন সম্পর্কিত গবেষণা  (*অনুগ্রহ করে, প্রতিwU প্রশ্নে শুধুমাত্র একটি বিকল্পে **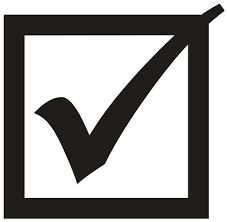** টিক দিন **Ges k~b¨¯’vb c~ib Kiæb**) | |
| --- | --- |
| **RbZvwZ¡K Z_¨ (1-15)**   1. **eqm**............eQi 2. w**j½-** K. cyiæl 🞎 L. gwnjv 🞎 3. **ag©-** K. gymwjg 🞎 L. wn›`y 🞎 M. Ab¨vb¨ ...............🞎 4. **Avcbvi eZ©gvb wkÿvel©** (Educational status/Academic Year)- B.Sc. Level (year)……….…………/   Masters............Semester   1. **Avcbvi পড়ার বিষয় (**Major of the study/Subject) ………………………. 2. **Avcbvi eZ©gvb wkÿv cÖwZôvb(wek¦we`¨vjq) -**   ……………………….   1. **Avcbvi eZ©gvb AvevwmK Ae¯’vb-**   K. cwiev‡ii mv‡_ 🞎  L. eÜz/n‡ji iæg‡gU‡`i mv‡_ 🞎  M. †g‡m iæg‡gU‡`i mv‡_ 🞎   1. **¯’vqx emev‡mi wVKvbv-** K. MÖvg 🞎 L. kni 🞎 †Rjvt................ 2. **evevi wkÿvMZ †hvM¨Zv-**   ক. cÖvwZôvwbK wkÿv ‡bB 🞎 L. cÖv_wgK 🞎 M. gva¨wgK 🞎  N. D”P gva¨wgK 🞎 O. ¯œvZK/ Gi Dc‡i   1. **gv‡qi wkÿvMZ †hvM¨Zv-**   ক. cÖvwZôvwbK wkÿv ‡bB 🞎 L. cÖv_wgK 🞎 M. gva¨wgK 🞎  N. D”P gva¨wgK 🞎 O. ¯œvZK/ Gi Dc‡i   1. **cwiev‡ii gvwmK Avq**(টাকা)   K. ১৫,০০০ টাকা **পর্যন্ত** 🞎  M. ১৬০০০ থেকে ৩০,০০০🞎  L. ৩০,০০০ এর উপরে 🞎   1. **gv‡qi ‡ckv-** ক. PvKzixRxex/Kg©Rxex 🞎 L. M„wnYx 🞎      1. **Avcwb ivbœv K‡ib/অভ্যাস আছে?**   K. n¨vu, memgq 🞎 M. n¨vu , Lye Kg 🞎  L. n¨vu, gv‡Sgv‡S 🞎 N. KLbB bv 🞎   1. **Avcbvi wK KLbI dzW cqRwbs/Lv‡`¨ welwµqv n‡qwQj?**   K. n¨vu 🞎 L. bv 🞎   1. **Avcbvi**/**cvwiev‡ii Rb¨ Lv`¨ µq/evRvi K‡ib?**   K. n¨vu, memgq 🞎 L. n¨vu, gv‡S gv‡S🞎 M. n¨vu ,Lye Kg🞎 N. KLbI bv 🞎  **খাদ্য নিরাপত্তা সম্পর্কিত বিভিন্ন বিষয়ের PP©v (1-14)**   1. **আপনি একটি KvwUs/**চপিং **†ev‡W© KuvPv gvsm UzK‡iv করতে চান Ges †m GKB †ev‡W© mewR KvU‡Z PvB‡j wb‡Pi †KvbwU mwVK Dcvq &n‡e?**   K. †evW©wU ‡hiKg Av‡Q †miKgB e¨envi Kiv 🞎  L. †evW©wU KvM‡Ri/Kvc‡oi †Zvqv‡j w`‡q gy‡Q †djv🞎  M. শাকসবজি কাঁটার জন্য †ev‡W©i Ab¨cvk e¨envi Kiv 🞎  N. Ab¨ GKwU চপিং †evW© e¨envi Kiv 🞎  O. Rvbv bvB 🞎   1. **Avcbvi iæg‡gU ev Avcwb hw` ivbœv Kiv Lvevi K‡qK N›Uv †`wi K‡i Lvb Zvn‡j wK K‡ib/Kiv DwPZ ?**   K. ‡iwd«Rv‡iU‡i ivwL, Zvici LvIqvi Av‡M cybivq Mig Kwi 🞎  L. Avjgvwi‡Z/†kj‡d †X‡K ivwL,LvIqvi Av‡M cybivq Mig Kwi🞎  M. gvB‡µvI‡qf I‡f‡b/DÂ Pzjvq ivwL 🞎  N. cybivq Mig Kiv nq bv🞎  O. VvÛv Pzjvq ivwL 🞎   1. **ivbœv Kivi Av‡M ev LvIqvi Av‡M nvZ †avqvi mwVK Dcvq †KvbwU?**   K. ïay VvÛv cvwb w`‡q nvZ †avqv 🞎  L. VvÛv cvwb I mvevb/n¨vÛ Iqvk w`‡q nvZ †avqv Ges gy‡Q †djv🞎  M. ‡Zvqv‡j /Kvco w`‡q gy‡Q †djv 🞎  N. LvIqvi Av‡M me mgq nvZ cwi¯‹vi Kiv nq bv🞎   1. **kvKmewR Ges djg~j wKfv‡e †avqv DwPZ e‡j Avcwb g‡b K‡ib ?**   K. wWUvi‡R›U/mvevb †gkv‡bv cvwb e¨envi K‡i 🞎  L. Mig cvwb e¨envi K‡i 🞎  M. (U¨v‡ci) Pjgvb VvÛv cvwb e¨envi K‡i 🞎  N. bigvj cvwb‡Z wfwR‡q †i‡L **†avqv** 🞎   1. **Lvevi ivbœv ev ˆZwii mgq wb‡Pi †KvbwU ¯úk© Kivi ci nvZ ay‡q †djv DwPZ?**   K. gyLgÛj 🞎 M. cwi¯‹vi cvÎ Ges KovB 🞎  L. Lvevi ˆZix‡Z e¨eüZ cvÎ 🞎 N. Dc‡ii †KvbwUB bq 🞎  **খাদ্য নিরাপত্তা m¤úwKZ Ávb (1৫-৩০)**   1. **wb‡Pi †KvbwU‡Z Campylobacter/ ÿwZKviK e¨K‡Uwiqv _vK‡Z cv‡i?**   K. Canned/wUbRvZ Lvev‡i 🞎 M. ‡d«k kvK-mewR 🞎  L. KvuPv A_ev Kg wm× gvQ/gvsm 🞎 N. Rvbv bvB 🞎   1. **wb‡Pi †KvbwU Listeria/ ÿwZKviK e¨K‡Uwiqv Øviv `~wlZ nIqvi m¤¢vebv A‡bK †ewk?**   K. Canned/wUbRvZ প্রসেসড Lvev‡i 🞎 M. ‡d«k kvK-mewR 🞎  L. KvuPv A_ev Kg wm× gvQ/gvsm/`ya 🞎 N. Rvbv bvB 🞎   1. **wb‡Pi †Kvb AbyRxe¸wj AwaKvsk Lv`¨RwbZ Amy¯’Zvi KviY?**   K. e¨vK‡Uwiqv 🞎 L. QÎvK 🞎 M. fvBivm 🞎  N. ciRxex 🞎 O. Rvwb bv 🞎   1. **wb‡Pi †Kvb e¨w³‡`i g‡a¨ Lv‡`¨ welwµqv nIqvi m¤¢vebv †ewk?**   K. eq¯‹ ‡jvK 🞎 M. wK‡kvi🞎  L. Mf©eZx gwnjv🞎 N. Rvbv bvB🞎   1. **evRvi Kivi mgq wd«wRs dzW/Lvevi KLb µq Kiv DËg ?**   K. ‡KbvKvUvi ïiæ‡Z 🞎 M. ‡h‡Kvb mgq 🞎  L. ‡KbvKvUvi ‡k‡l 🞎 N. Rvbv bvB 🞎 | 1. **KuvPv gvsm UzK‡iv Kivi ci KvUvi/PvKzwU cybivq e¨envi Ki‡Z PvB‡j wb‡Pi †KvbwU mwVK Dcvq &n‡e?**   K. KvUvi/PvKzwU ‡hiKg Av‡Q †miKgB e¨envi Kiv 🞎  L. KvUvi/PvKzwU VvÛv cvwb w`‡q ay‡q †djv 🞎  M. KvUvi/PvKzwU KvM‡Ri/Kvc‡oi †Zvqv‡j w`‡q gy‡Q †djv 🞎  N. wWUvi‡R›U Ges Mig cvwb w`‡q cwi®‹vi Kiv 🞎   1. **GKwU †iwd«Rv‡iU‡i wZbwU ‡kjd/ZvK Av‡Q; †Kvb Zv‡K KvuPv gvsm ivLv DwPZ e‡j Avcwb g‡b K‡ib?**   K. kxl©/me‡P‡q Dc‡ii Zv‡K 🞎 M. wb‡Pi Zv‡K 🞎  L. gv‡Si Zv‡K 🞎 N. †h †Kvb Zv‡K; GUv †Kvb e¨cvi bv 🞎   1. **GKRb gvby‡li nv‡Z hw` ÿZ _v‡K Zvn‡j Lvevi ivbœv/cwi‡ekb Kiv wK wbivc` n‡e?**   K. n¨vu; hw` ÿZ¯’vb evuav‡bv _v‡K 🞎  L. n¨vu; hZÿY ch©šÍ ÿZ msµwgZ bv nq 🞎  M. n¨vu; hZÿY nv‡Z Møfm civ nq 🞎  N. G‡Kev‡iB bv 🞎   1. **Lvevi ivbœv ev cÖ¯‘Z Kivi mgq wKfv‡e cixÿv K‡ib †h ivbœv h‡_ô n‡q‡Q?**   K. Lvev‡ii is †`‡L I ¯^v` wb‡q 🞎 M. cvwZ‡ji †K‡›`ªi ZvcgvÎv ‡g‡c🞎  L. Lvev‡ii `„pZv ev MvpZ¡ ‡`‡L🞎 N. ivbœvi mgq cwigvc K‡i🞎   1. **Aewkó/**রেখে দেয়া **Lvevi** কতক্ষণ **ধরে Mig করেন?**   K. dzUšÍ/ev®ú ïiæ nIqv ch©šÍ Mig Kiv🞎  L. Abygvb K‡i GKUv wbw`ó ZvcgvÎvq Mig Kiv 🞎  M. Kÿ ZvcgvÎvq (25^0^ †mjwmqvm) Mig Kiv 🞎  N. cybivq Mig Kivi cÖ‡qvRb †bB 🞎  O. Rvbv bvB 🞎   1. **nvZ †avqvi mgq KZÿb hver nvZ সাবান দিয়ে ঘষেন ?**   K. 10 †m‡KÛ 🞎 L. 20 †m‡KÛ 🞎 M. 30 †m‡KÛ 🞎  N. 40 †m‡KÛ 🞎 O. Rvbv bvB 🞎   1. **Lvevi ivbœv ev cÖ¯‘Z Kivi mgq wK Ry‡qjvwi/Mqbv Ly‡j iv‡Lb?**   K. n¨vu, memgq 🞎 L. bv 🞎 M. n¨vu, gv‡S gv‡S 🞎 N. cÖ‡hvR¨ bq 🞎   1. **wb‡Pi †KvbwU wd«wRs Kiv gvsm Mjv‡bvi me‡P‡q fv‡jv Dcvq ?**   K. ‡iwd«Rv‡iU‡i /bigvj wd«‡R †i‡L 🞎  L. Kÿ ZvcgvÎvq gvsm KvUvi †ev‡W©/Lvwj cv‡Î †i‡L 🞎  M. VvÛv cvwbi c¨v‡KU/cv‡Î †i‡L 🞎  N Pjgvb U¨v‡ci cvwb‡Z 🞎  O. Rvbv bvB 🞎   1. **A‡bK mgq we`¨yr P‡j hvIqvi ci Avcbvi wd«R †_‡K gvsm ev gvQ Mjv ïiæ Ki‡j Avcwb wK Ki‡eb?**   K. wd«R †_‡K †ei K‡i †d‡j †`qv 🞎  L. mwVK Dcv‡q †m¸‡jv ZLwb ivbœv Kiv 🞎  M. wm×všÍ †bqvi Av‡M MÜ Ges is †`‡L †bqv 🞎  N. we`¨yr Avmvi mv‡_ mv‡_B cybivq wd«wRs Kiv Ges k³ nIqvi ci ivbœv Kiv 🞎 ***(Aci c„ôv †`Lyb)***   1. **ivbœv Kiv Lvev‡ii Af¨šÍixY ZvcgvÎv KZ n‡j IB Lvevi‡K wbivc` e‡j g‡b Kiv nq?**   K. 54^0^ †mjwmqvm 🞎 L. 60^0^ †mjwmqvm 🞎 M. 66^0^ †mjwmqvm 🞎  N. 74^0^ †mjwmqvm 🞎 O. Rvbv bvB 🞎   1. **wbivc` Dcv‡q wWg fvRv †L‡Z PvB‡j wb‡Pi †KvbwU mwVK Dcvq n‡e?**   K. mv`v Ask Ges Kzmyg DfqB k³ 🞎  L. mv`v Ask Ges Kzmyg DfqB Avav k³🞎  M. mv`v Ask k³ Ges Kzmyg Avav k³🞎  N. mv`v Ask k³ Ges Kzmyg Zij🞎  O. Rvbv bvB 🞎   1. **Lvev‡i Salmonella/ÿwZKviK e¨K‡Uwiqv cÖwZ‡iv‡ai wbivc` Dcvq †KvbwU n‡Z cv‡i?**   K. Lvevi cy‡ivcywi Mig K‡i 🞎  L. Lvevi wZb w`‡bi †ewk wd«wRs K‡i 🞎  M. †miKg Lvevi ivbœv Kiv wbivc` n‡e bv 🞎  N. Rvbv bvB 🞎   1. **wb‡Pi †Kvb DcmM© _vK‡j Ab¨‡`i Rb¨ Lvevi ivbœv Kiv DwPZ bq?**   K. Wvqwiqv, R¡i, Mjv e¨v_v A_ev d¬z n‡j 🞎 M. gv_ve¨v_v n‡j 🞎  L. Z¡‡Ki GjvwR© _vK‡j 🞎 N. Dc‡ii me¸‡jv🞎   1. **‡iwd«Rv‡iU‡i Lvevi msiÿ‡Yi wb‡`©wkZ ZvcgvÎv KZ?**   K. **-**4 ^0^ †mjwmqvm 🞎 M. 4 ^0^ †mjwmqvm 🞎  L. 12 ^0^ †mjwmqvm 🞎 N. Rvbv bvB 🞎   1. **wWc wd«‡R Lvevi msiÿ‡Yi wb‡`©wkZ ZvcgvÎv KZ?**   K. **-**18 ^0^ †mjwmqvm 🞎 M. 18 ^0^ †mjwmqvm 🞎  L. 0^0^ †mjwmqvm 🞎 N. Rvbv bvB 🞎   1. **wb‡Pi †KvbwU †L‡j dzW cqRwbs/Lv‡`¨ welwµqv n‡Z cv‡i?**   K. ‡iwd«Rv‡iUi †_‡K †ei Kiv dj †L‡j  L. KvuPv/Kg wm× wWg †L‡j 🞎 N. (L Ges M DfqB) 🞎  M. Kg wm× Kiv gvsm †L‡j 🞎 O. Ab¨vb¨ 🞎   1. **wd«wRs Kivi gva¨‡g Lvev‡ii ÿwZKi RxevYy wbg©~j/AcmviY Kiv hvq?**   K. mwVK 🞎 L. fzj 🞎 M. Rvbv bvB 🞎   1. **wb‡Pi †KvbwU dzW cqRwbs/Lv‡`¨ welwµqv cÖwZ‡iv‡ai me‡P‡q ¸iæZ¡c~Y© Dcvq?**   K. ivbœvN‡i cÖwZ mßv‡n RxevYybvkK wQwU‡q cwi®‹vi Kiv 🞎  L. evwm ev †i‡L †`qv Lvevi cwinvi Kiv 🞎  M. Lvevi cwi‡ek‡b †`wi n‡j †iwd«Rv‡iU‡i ivLv 🞎  N. Lvevi LvIqv ev ivbœvi Av‡M fv‡jvfv‡e nvZ †avqv 🞎  O. Dc‡ii me¸‡jv 🞎   1. **wb‡Pi †KvbwU ivbœvN‡ii evmb‡Kvmb †avqvi mwVK Dcvq?**   K. K‡qKN›Uv cvwb‡Z wfwR‡q wWUvi‡R›U/QvB w`‡q †avqv I ïKv‡bv 🞎  L. LvIqvi ci ciB wWUvi‡R›U/ QvB w`‡q †avqv I ïKv‡Z †`qv 🞎  M. LvIqvi ciciB wWUvi‡R›U/QvB w`‡q †avqvI†Zvqv‡j w`‡q ïwK‡q †djv 🞎  N. ¯^qswµq wWmIqvmvi w`‡q †avqv I ïwK‡q †djv 🞎   1. **‡KvbwU ivbœvNi Ges Pzjv cwi®‹vi Kivi mwVK Dcvq?**   K. wWUvi‡R›U/mvevb Ges VvÛv cvwb w`‡q cwi®‹vi Kiv Zvici m¨vwbUvBRvi e¨envi Kiv 🞎  L. m¨vwbUvBRvi e¨envi Kiv Zvici cvwb w`‡q †avqv 🞎  M. cvwb w`‡q eªvk Kiv Zvici m¨vwbUvBRvi e¨envi Kiv 🞎  N. ïay cvwb w`‡q cwi®‹vi Kiv 🞎 |

-^-^- **Avcbvi g~j¨evb mg‡qi Rb¨ AmsL¨ ab¨ev`**-^-^-
