# Supplementary material for: Food safety and handling knowledge and practices among university students of Bangladesh: A cross-sectional study
Source: Heliyon. 2022 Nov 30;8(12):e11987. doi: 10.1016/j.heliyon.2022.e11987 (PMC9720042; doi:10.1016/j.heliyon.2022.e11987)
Supplement: Questionnaire (English Version).docx [file mmc2.docx]

**Food safety and handling knowledge and practices among university students of Bangladesh: a cross-sectional study**

**Section 1: Demographic characteristics of the study population**

|  | Age | …………………Years |
| --- | --- | --- |
|  | Gender | 1. Male 2. Female |
|  | Religion | 1. Muslim   B. Hindu  C. Others |
|  | **Your current Educational status** | 1. B.Sc. Level (year)………. 2. Masters.........Semester |
|  | **Major of the study/Subject** | …………………………………. |
|  | **Present Educational Institution (University)** | ……………………………………………….. |
|  | Residential Status | 1. With Family |
|  |  | 1. With Friends/Roommates in Hall |
|  |  | 1. In mess with roommates |
|  | Residential Area | 1. Rural 2. Urban District:.................. |
|  | **Father’s Education** | 1. No formal education 2. Primary 3. Secondary 4. Higher Secondary 5. Bachelor and/or above |
|  | **Mother’s Education** | 1. No formal education 2. Primary 3. Secondary 4. Higher Secondary 5. Bachelor and/or above |
|  | **Family Monthly Income (BDT)** | 1. Upto 15000 2. 16000 to 30000 3. Above 30000 |
|  | **Mothers employment status** | 1. Employment/works 2. Housewife |
|  | Cooking habit | 1. yes, all times 2. yes, Sometimes 3. yes, rarely 4. never |
|  | **Personal Food poisoning experience** | 1. Yes 2. No |
|  | **Involvement in food purchasing for personal or family** | 1. yes, all times 2. yes, Sometimes 3. yes, rarely 4. never |

**Section 2: Food handling practices questions.**

| Sl.  No. | Questions/statements | Multiple-choice responses |
| --- | --- | --- |
|  | You cut meat on a chopping board and now you wants to cut vegetables. Of the following, which one do you practice? | 1. Use the board as it is. 2. You wipe the board off with a paper towel/cloths 3. Use the other side of the chopping board to cut vegetables 4. Use another chopping board to cut vegetables 5. Don’t know |
|  | When you cut raw meat and need to use the knife again, what do you do? | 1. You reuse the knife as it is 2. You rinse the knife with cold water 3. You wipe the knife with a cloth/paper towel 4. You wash the knife with soap and hot water |
|  | A refrigerator has three shelves, on which shelf do you place raw meat? | 1. Top shelf 2. Middle shelf 3. Bottom shelf 4. Does not matter |
|  | Do you handle food if you have a wound on the back of your hand? | A. Yes, as long as the wound has a bandage on it  B. Yes, as long as the wound is not infected  C. Yes, as long as gloves are worn  D. Not at all |
|  | How do you check that food is sufficiently cooked? | 1. By seeing the food color/By taking taste 2. Density of Juice content/concentration of food 3. By checking the central temp. of cooking pot 4. Measuring the cooking time |
|  | How long do you heat Leftover foods? | 1. Until they are boiling hot 2. Heat it to the temperature you prefer 3. Just until they are at least at room temperature or 25^0^C 4. Reheating is not necessary 5. Don’t know |
|  | While washing your hands, how long do you rub them with soap? | 1. 10 seconds 2. 20 seconds 3. 30 seconds 4. 40 seconds 5. Don't know |
|  | Do you take off the jewelry when preparing food? | 1. Yes 2. No 3. Yes, sometimes 4. Not applicable |
|  | Of the following, how do you thaw raw meat? | 1. Thaw In refrigerator 2. Thaw on chopping/cutting board (25 0 C / room temperature) 3. Thaw in cold water in sealed package/pot 4. Thaw In running water 5. Don't know |
|  | In case your electricity went off and the meat, chicken, and/or seafood in your freezer thawed and felt warm, what do you do? | 1. Throw them away 2. Cook them right away 3. See how they smell or look before deciding what to do 4. Immediately re-freeze until future consumption |
|  | If your roommate or you are going to be several hours late for a hot meal, where do you leave the meal? | 1. Store it in the refrigerator and reheat it when the person is ready to eat it 2. Store it in on the kitchen counter until the person is ready to eat it 3. Store it in a warm oven until the person is ready to eat it Not reheat again 4. Store it in a cool oven until the person is ready to eat it 5. Don't know |
|  | How do you wash your hands before starting preparing food or eating? | 1. Cold Water only 2. Wash hand With soap/hand wash and cold water 3. Wipe with a towel or dish cloth 4. I don’t clean them at all |
|  | You wash fruits and vegetables by using: | 1. Water and soap 2. Hot water 3. We wash them under cold running water 4. Using Normal water |
|  | After touching which of the following do you wash your hands during the course of preparing food? | 1. Face 2. Clean cooking utensils/cooking pot 3. Clean utensils 4. None of the above |

Section 3: **Food safety knowledge questions**

| Sl.  No. | Questions/statement | Multiple-choice responses |
| --- | --- | --- |
|  | Campylobacter bacteria are most likely associated with which food? | A. Canned food  B. Raw or undercooked meat/fish  C. fresh vegetables  D. Don’t know |
|  | Which of the following is most likely to become contaminated with Listeria? | A. Canned food  B. Raw or undercooked meat/fish  C. Fresh vegetables  D. Don’t know |
|  | The microorganisms that cause most of food-borne illnesses are: | 1. Bacteria. 2. Fungi. 3. Viruses. 4. Parasites. 5. Don’t know |
|  | Which of these individuals are LEAST likely to get food poisoning? | 1. Old people. 2. Pregnant women. 3. Teenagers. 4. Don’t know |
|  | When is the best time to purchase frozen food when shopping? | 1. At the beginning of the shopping time 2. At the end of the shopping time 3. Whenever, does not matter 4. Don't know |
|  | All foods are considered safe when cooked to an internal temperature | 1. 54 ° C 2. 60 ° C 3. 66 ° C 4. 74 ° C 5. Don't know |
|  | Which is the safest way to get fried egg? | 1. Solid albumen and yolk 2. Semi-solid albumen and yolk 3. Solid albumen and semi-solid yolk 4. Solid albumen and liquid yolk 5. Don’t know |
|  | How to prevent salmonella poisoning? | 1. Fully heat food 2. Freeze food for more than 3 days 3. Those food will not safe for cooking 4. Don't know |
|  | People with which of the following symptoms should not cook for others? | 1. Diarrhea, Fever, Sore throat or Flu 2. Skin allergies 3. Headache 4. All the Above |
|  | What is the maximum refrigerators temperature should be to preserve the safety of foods? | 1. -4 ° C 2. 12°C 3. 4 °C 4. Don't know |
|  | What is the recommended temperature for freezers? | 1. -18°Cd 2. 0°C 3. 18 °C 4. Don't know |
|  | You can get food poisoning from eating which of the following? | 1. Fruits taken out of the refrigerator immediately 2. Raw or undercooked eggs 3. Raw or undercooked meat 4. Both B & C 5. Others |
|  | Freezing Kills harmful germs in food | 1. Right 2. Wrong 3. Don't know |
|  | Which is the most important for preventing food poisoning? | 1. Use detergent to disinfect kitchen countertop and stove weekly 2. Avoid eating leftovers 3. Keep food refrigerated until it is time to serve them 4. Washing hands properly before eating 5. Don't know |
|  | Of the following, which do you think is the correct way to wash dishes? | 1. Soak in water, after several hours, wash with the same water using detergent/Ash/Soap 2. Wash immediately after meal using detergent/Ash/Soap and wipe off 3. Wash immediately after meal using detergent/Ash/Soap and wipe off by towel 4. Wash with automatic dish washer |
|  | Which of the following scenario for cleaning kitchen counters and stoves are the best? | 1. Brush with Soap/detergent and water, then use sanitizer 2. Using Sanitizer, then water 3. Brush with water, then sanitizer 4. Water, then drying |

**Thank you very much for your valuable time**
